# Teaching the Retropubic Midurethral Sling Using a Novel Cadaver and Model-Based Approach

**DOI:** 10.7759/cureus.1214

**Published:** 2017-05-02

**Authors:** Sallie Oliphant, Eliza Beth Littleton, Gabriella Gosman, Gary Sutkin

**Affiliations:** 1 Department of Obstetrics and Gynecology, University of Arkansas for Medical Sciences; 2 University of Pittsburgh-School of Medicine; 3 Division of Reproductive Endocrinology and Infertility, University of Pittsburgh-School of Medicine; 4 Department of Obstetrics and Gynecology, University of Missouri Kansas City (UMKC)

**Keywords:** midurethral sling, surgical teaching, surgical simulation

## Abstract

**Objective:**

To measure the impact of a model-based teaching program on resident comfort and skill with retropubic midurethral sling (MUS).

**Study design:**

Residents were assessed before and after a retropubic MUS teaching session, which included a brief lecture and three interactive teaching stations (cadaver pelvis, retropubic MUS pelvic model, cystoscopy model). Self-assessment measures included MUS-related visual analog scale (VAS), Likert, and open-ended questions. Objective assessment measures were used to score blinded videos of trocar passage on a pelvic model, including a modified objective structured assessment of technical skills (mOSAT) and a retropubic MUS-specific checklist of surgical steps. Emerging themes from the open-ended questions were identified using grounded theory; analysis ceased once theme saturation was achieved.

**Results:**

Twenty-five of 37 total residents participated in the training session and 24 participated in this study. Following training, VAS scores, Likert scores, and qualitative analysis indicated greater resident comfort with performing retropubic MUS, with relevant anatomy, and with trocar passage. Residents demonstrated improvement in model trocar passage post-training, with a rise in mOSAT score (47% to 65%; p = .01) and a rise in checklist score (61% to 75%; p = .11). Residents expressed discomfort due to inexperience with MUS, concern regarding trocar passage, and worry over potential complications. Residents reported feeling more prepared to perform MUS after the session. They stressed the importance of repetition and a comfortable learning environment for surgical training, and praised the “hands-on” training session.

**Conclusion:**

We demonstrate success using a short, single-session, hands-on group training session to improve comfort and skill with retropubic MUS.

## Introduction

Surgical simulation is a valid and increasingly popular method to teach surgical skills [1-3]. Use of a simulation model and the presentation of focused content knowledge prior to real-life surgery provides an opportunity for early surgical skill acquisition, allowing the trainee to focus on higher level skills during subsequent intra-operative experiences [4]. Gynecologic surgical training using simulation to afford maximum learning during surgical cases is increasingly relevant as trainee work hours decline and hospitals focus on reducing operating room times.

Midurethral sling (MUS) is a surgical procedure performed to treat stress urinary incontinence and has become the “gold-standard” for surgical management of this common complaint [5]. A study of recent graduates of a large academic Obstetrics and Gynecology (Ob/Gyn) residency training program found that few residents continue to perform MUS in practice, despite exposure and training during residency [6]. Prior data from our institution has shown that learners experience significant anxiety with the skill of trocar passage during MUS [7]. Trocar passage in the training environment has been associated with high rates of bladder perforation, particularly with vaginal trocar introduction [8]. The MUS technique is unlike most other gynecologic surgical procedures as it involves blind instrument passage in a complex three-dimensional anatomic space. We feel that MUS is a procedure within the purview of the generalist gynecologist, thus residents should achieve competence with MUS during residency training. The goal of this study was to introduce a cadaver and model-based teaching program to our residents and measure its impact on resident comfort and skill with passage of retropubic trocars in a MUS model.

## Materials and methods

**Figure 1:**
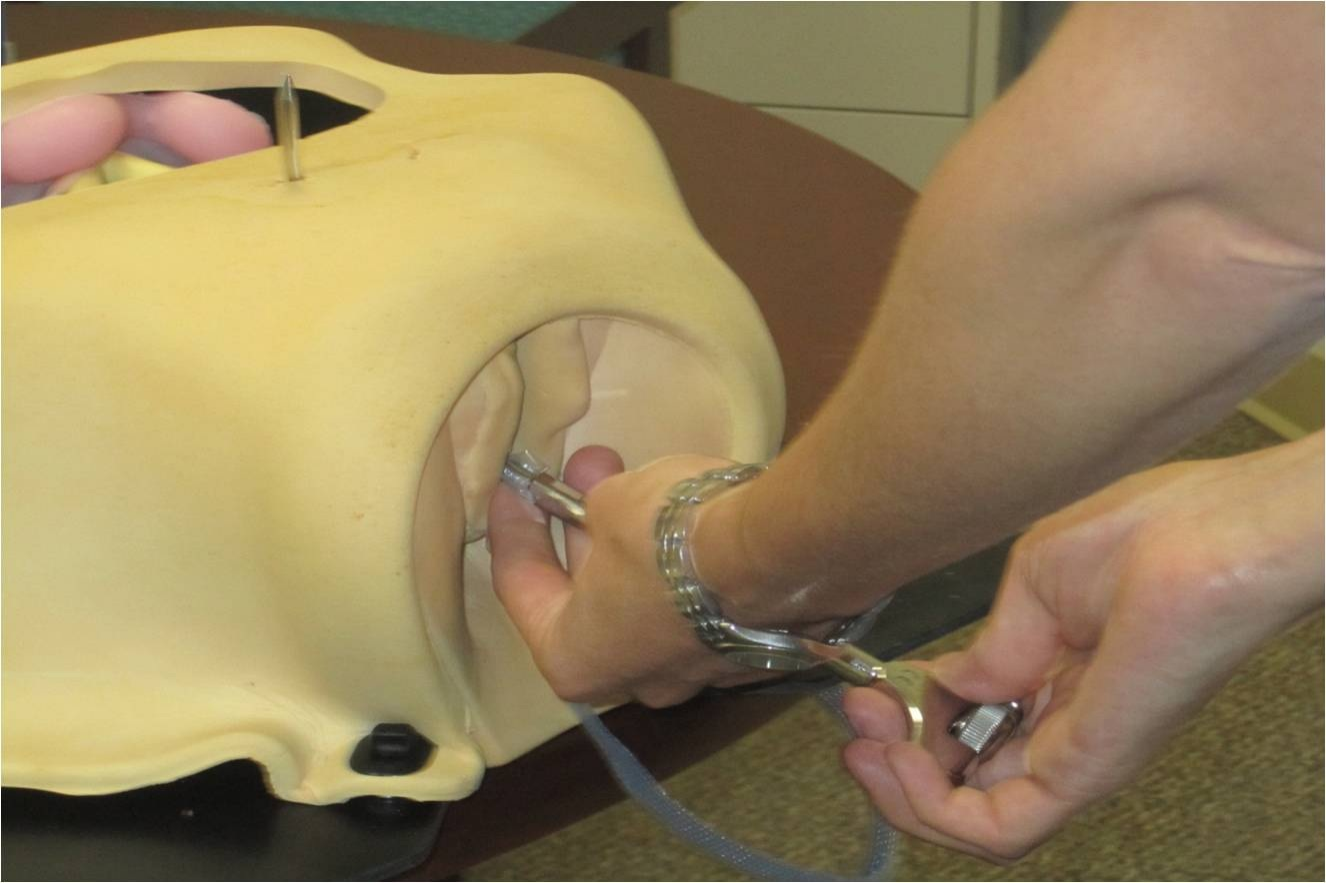
Pelvic model for retropubic trocar passage

**Figure 2:**
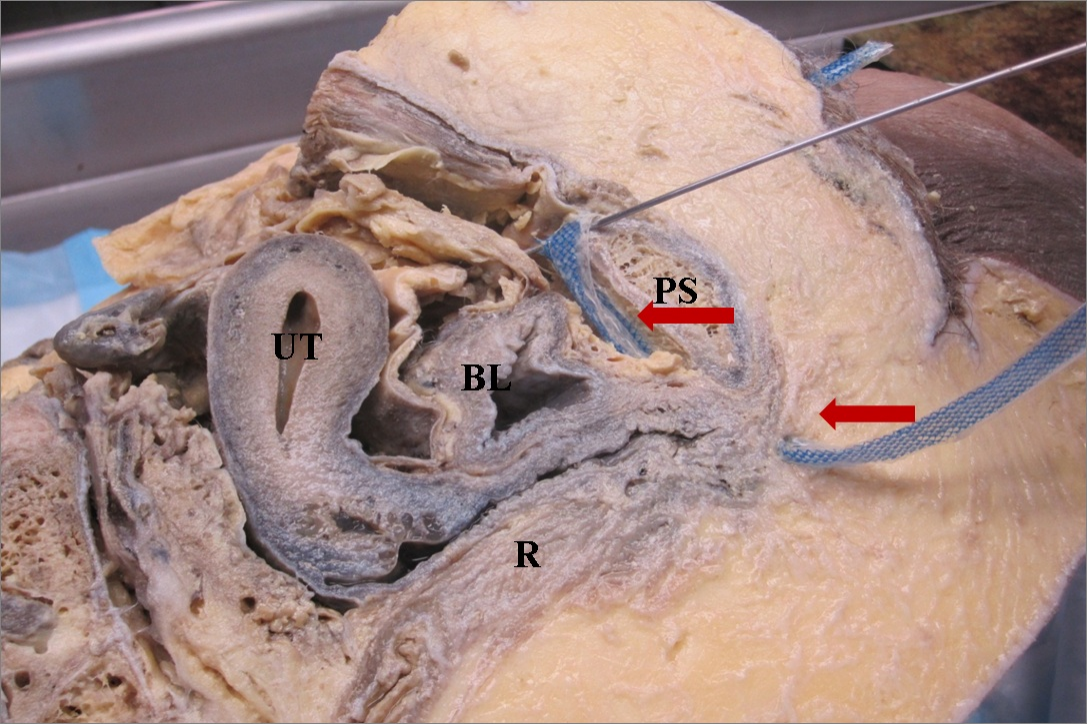
Hemisected cadaver pelvis demonstrating correct trocar placement Arrows: Trocar/mesh track; BL: Bladder; PS: Pubic symphysis; R: Rectum; UT: Uterus.

**Figure 3:**
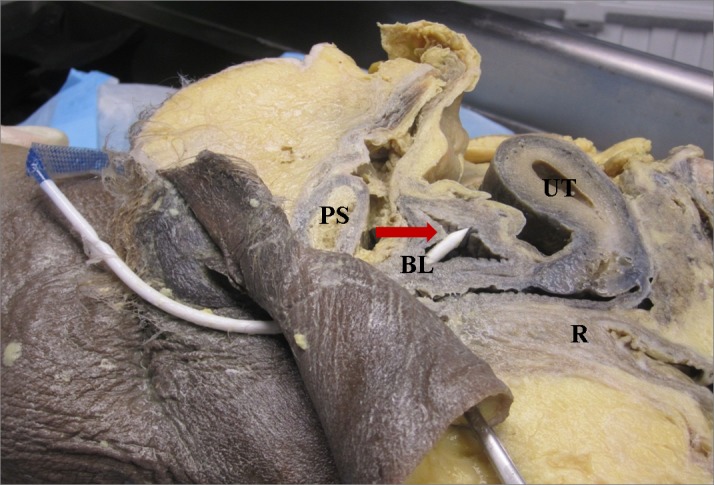
Hemisected cadaver pelvis demonstrating trocar bladder perforation Arrows: Trocar/mesh track; BL: Bladder; PS: Pubic symphysis; R: Rectum; UT: Uterus.

Following IRB approval, we offered enrollment to Ob/Gyn residents (Post-graduate year (PGY) 1-4) at the University of Pittsburgh, Magee-Women’s Hospital, Pittsburgh, Pennsylvania, USA, who were participating in a scheduled teaching session on MUS. Ob/Gyn residents at our institution spend three weeks in the PGY-2 year and another six weeks in the PGY-3 year rotating on the Urogynecology clinical service, during their four years of residency training. All participants signed consent forms prior to study participation. The training session, proctored by our Urogynecology Division, consisted of a 30 minute lecture on MUSs followed by rotation through three hands-on teaching stations. The lecture reviewed indications for MUS, various sling types and surgical approaches, relevant anatomy, and recognition and management of potential complications. Following the lecture, residents were divided into small groups and rotated through each of the teaching stations, spending approximately 20 to 30 minutes at each station. Teaching stations were proctored by two attending- and two fellow-level Urogynecologists and included: 1) retropubic trocar passage on pelvic models; 2) identification of trocar bladder injury on cystoscopy model; and 3) anatomy review with a hemisected, prosected cadaver pelvis. We used two retropubic trocar passage models (Figure 1), available through Limbs and Things (Bristol, UK), with Gynecare TVT sling kits (retropubic with vaginal trocar introduction). A cadaver pelvis was hemisected and prosected to expose retropubic anatomy. One side demonstrated correctly placed MUS trocar/mesh (Figure 2), while the other demonstrated trocar perforation of the bladder (Figure 3). Thus our education intervention consisted of a single session that comprised of a didactic lecture and station-based training, which included a variable number of trocar practice passes using a retropubic MUS model. In the training session, residents could pass the trocar as many times as they deemed necessary for learning and comfort with the procedure.

**Figure 4:**
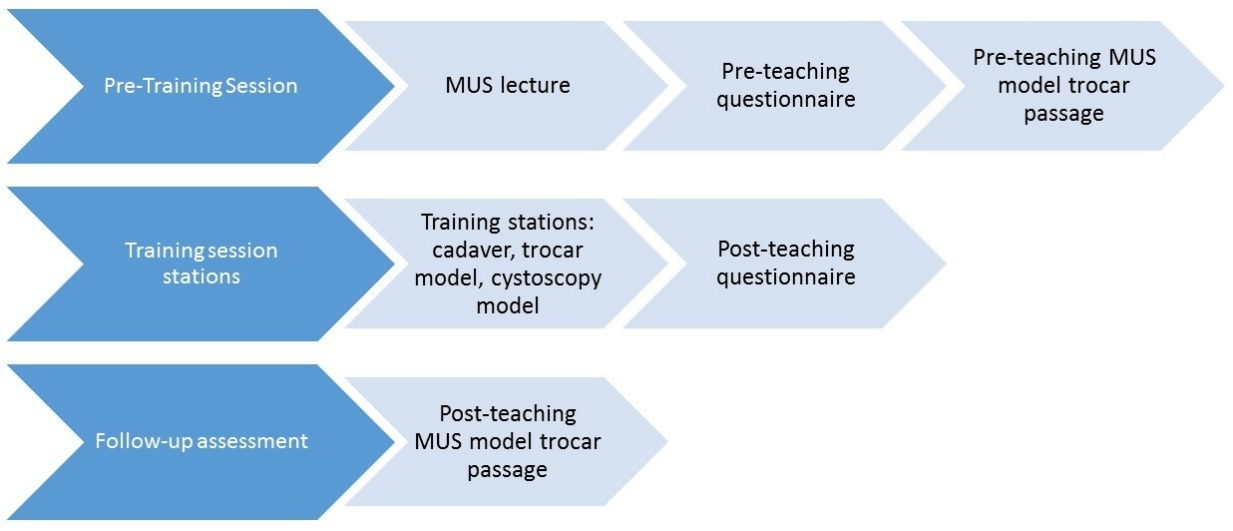
Study flow diagram MUS: Midurethral sling.

Prior to the session residents had been instructed to self-review a video module (provided by Gynecare) and textbook chapter [9] covering the retropubic MUS procedure. These materials were available to all residents in the resident library in the month prior to the teaching session. These materials mirror the usual expected resident preparation prior to performing the MUS in the operating room. At the start of the training session, participants were filmed passing a retropubic MUS trocar on a pelvic model and completed a pre-teaching questionnaire. Immediately following the training session, residents completed the post-teaching questionnaire. To assess skill retention, residents were filmed performing model trocar passage again approximately one month after the training session. This follow-up session required the residents to schedule a short filming session on the pelvic model during their free time. Please see Figure 4 for a flow diagram of the study design. Study questionnaires included questions regarding training level, operative experience with MUS, comfort with MUS anatomy, procedure and complications, surgical training, and plans to perform MUS in practice using both 10 centimeter (cm) visual analog scale (VAS) and Likert-type responses. Free response questions addressed aspects of learning the MUS and elicited feedback on the teaching session. Questionnaires were anonymous and de-identified. A single trocar pass per resident (1-2 minutes’ duration) was filmed at both the pre- and post-teaching timepoints for the purpose of blinded review and scoring. Videos were blinded (hands only, no audio) and scored by two independent Urogynecologists, using a modified objective structured assessment of technical skills (mOSATS) scale and a MUS-specific checklist of surgical steps (Table 1), both developed by the authors for this project [10]. OSATS were modified to exclude those items not able to be scored from a surgical step-specific video (ex. knowledge of procedure). If video footage prevented interpretation (ex. angle of image obscured view) for a particular sub-category or step on either mOSATS or checklist, the resident was not scored for this particular sub-category or step. mOSATS and checklist scores were calculated as percentages.

 

**Table 1:** MUS-specific checklist of surgical steps and modified objective structure assessment of technical skills (mOSATS) scale * Checklist items which were unable to be assessed due to videography angle were omitted from total percentile score. MUS: Midurethral sling; OSATS: Objective structured assessment of technical skill.

MUS Checklist	Scoring: 0-8 out of 8*
(1) Correct hand placement in preparation for trocar pass. One hand on T/handle, other hand on shaft.	Yes (1); No (0)
(2) Placed trocar into dissection tunnel gently (no obvious force noted).	Yes (1); No (0)
(3) Correct angle of trocar alignment (toward theoretical ipsilateral shoulder).	Yes (1); No (0)
(4) Catheter guide deviated to ipsilateral side for passage by assistant.	Yes (1); No (0)
(5) Once behind the bone, trainee dropped handle to continue pass close behind symphysis, following the curve of the bone.	Yes (1); No (0)
(6) Trainee used two hands to stabilize trocar during entire passage (until past fascia).	Yes (1); No (0)
(7) Angle of passage correct throughout (handle parallel to floor).	Yes (1); No (0)
(8) Controlled exit through skin using own hand or assistant’s hand.	Yes (1); No (0)
Modified OSATS	Scoring: 3-15 out of 15
(1) Time and motion	1 (worst) to 5 (best)
(2) Instrument handling	1 (worst) to 5 (best)
(3) Use of assistants	1 (worst) to 5 (best)

For quantitative analysis residents were grouped as those who had completed the PGY-3 Urogynecology rotation vs those who had not, since at our training program the PGY-3 rotation is the mainstay of Urogynecology clinical and surgical experience. Baseline data were analyzed using medians and frequencies where appropriate. Data were assessed for normality. The Wilcoxon Signed Rank test was used to compare the change in responses and scores following training and Mann-Whitney U test was used for group comparisons. Interrater reliability was measured using Pearson’s correlation coefficient. p-value of less than 0.05 was considered significant. Resident surveys were reviewed by three investigators (SO, EL, GS) and emerging themes were identified from the free responses in order to augment the quantitative data. Generation of themes ceased once theme saturation was achieved [11]. To further explore identified themes we attempted to conduct a series of individual, audio-taped interviews with trainees, but were unable to recruit participants for these interviews, estimated to take approximately 30 minutes to complete.

## Results

Twenty-five of the 37 residents at our institution attended the teaching session, with 65% (24/37) participating in this study. Participants included 65% junior level residents (PGY-1: n = 8, PGY-2: n = 7) and 35% senior level residents (PGY-3: n = 4, PGY-4: n = 4). Data regarding training level was missing for one participant. Many residents (41.7%) had not yet rotated on the Urogynecology service, with only 37.5% having completed the PGY-3 Urogynecology rotation and 16.7% having completed the PGY-2 Urogynecology rotation. Residents were queried about their cumulative surgical experience with MUSs. The median number of MUSs performed by all residents was 3 (IQ range 3-13). For those residents who had completed the PGY-2 and PGY-3 Urogynecology rotations, the median number of MUSs performed was 17 (IQ range 8-27). Six residents (25%) reported no prior MUS surgical experiences. Only 56.6% of MUSs performed were retropubic; the others were transobturator (35.5%) or single incision (7.7%).

Table 2 outlines resident median VAS scores pre- and post-teaching. Subanalysis by the group based on the status of completion of the PGY-3 Urogynecology rotation suggested more improvement occurred for the less experienced learners. Those who had not completed the PGY-3 rotation had significant improvement in median VAS change scores post-teaching compared to those who had completed the rotation in the areas of comfort performing MUS (2.4 vs 0.5, p = .007), comfort with steps of trocar passage (2.8 vs 0.7, p = .002), and comfort with retropubic anatomy (1.4 vs 0.3, p = .046). No differences were seen in median VAS change scores between subgroups for comfort with complication recognition, degree of preparedness, or plans to perform MUS. Prior to the teaching session, residents endorsed feeling generally satisfied with their overall pelvic surgical training by Likert scale response, with pre-teaching responses of 61% “satisfied” or “somewhat satisfied.” Interestingly, following the teaching session, this satisfied group increased to 82%. Just eight percent of residents were “somewhat dissatisfied” with both pre- and post-teaching. Prior to the training session, resident open-ended responses reflected the lack of experience with MUS, discomfort with trocar passage, and discomfort with potential complications. Following training, residents praised the “hands-on” nature of the session and stressed the importance of repetition and a comfortable learning environment for surgical teaching. There were no discernable differences in themes based on training or experience level. Table 3 (please see Appendix) lists identified themes with representative resident responses.

**Table 2:** Pre- and post-teaching visual analog scale (VAS) scores °All questions were marked on a 10 cm scale, with higher numbers reflecting more comfort, preparedness, etc. Representative prompt phrasing: “Make an X on the line to indicate how comfortable you would feel performing a retropubic midurethral sling independently”. *Scores are reported as median (IQ range). Units are centimeters. ^Wilcoxon signed-rank test

VAS assessment°	Pre-score° n = 24	Post-score* n = 23	p-value^
(1) Comfort performing a retropubic midurethral sling independently	2.4 (0.5, 5.3)	5.2 (3.0, 7.3)	<0.001
(2) Comfort with steps of retropubic trocar passage	3.2 (1.4, 5.8)	5.9 (2.9, 6.9)	0.001
(3) Comfort with anatomy of retropubic space	4.4 (2.0, 5.9)	6.7 (5.3, 7.4)	<0.001
(4) Comfort with ability to recognize a complication of retropubic midurethral sling	4.3 (2.4, 6.3)	5.4 (3.3, 6.8)	0.094
(5) Degree that surgical training has prepared trainee to perform retropubic midurethral sling	3.7 (1.6, 6.1)	5.2 (2.7, 7.0)	0.008
(6) How likely trainees will be to perform midurethral sling in practice (if they were to pursue generalist practice)	5.8 (1.7, 8.0)	7.4 (2.1, 8.7)	0.145

Residents demonstrated improvement in model trocar passage following the teaching session, with a rise in mOSATS median score from 47% to 65% (p = .008). The increase in sling checklist median score from 61% to 75% was not statistically significant (p = .114). Interrater reliability, as measured by Pearson’s correlation coefficient, was high for the checklist scores (r = .716) and moderate for the mOSATS (r = .489). No differences in mOSATS score change or sling checklist score change were seen when comparing groups based on the status of completion of PGY-3 Urogynecology rotation. Overall participation and follow-up rates were higher in the junior resident (PGY-1, PGY-2) group. Initial participation rate was high, but unfortunately only half of those residents initially participating attended the post-test MUS trocar model session, representing just 32% (12/37) of all residents. There were no significant differences between those participants who did and did not complete the follow-up assessment with regard to training level, sling surgical experience, pre-teaching or post-teaching self-assessment scores, or satisfaction with the training session. Similarly, we were unable to generate additional qualitative data due to lack of trainee recruitment for our proposed half-hour individual interviews.

## Discussion

In a relatively novice resident group, we demonstrate success in using a short, single session, hands-on group training session to improve resident comfort and skill with a MUS model. Both time constraints from duty hour restrictions and declining operative volume provide training challenges for today’s Ob/Gyn residents. Simulation training has been widely adopted for laparoscopic and robotic training in gynecology, but has been underutilized in the mastery of vaginal procedures [12-13]. Prior work has shown simulation to be effective for teaching MUS at the resident level [14]. This study is unique in that we utilized both quantitative and qualitative assessment to demonstrate improved MUS comfort and skill following training with a pelvic model and prosected cadaver pelvis.

In our study, residents reported a median of 17 MUS at the conclusion of their PGY-3 urogynecology rotation, which is slightly higher than numbers reported at other large academic training programs [7]. Though the majority of these reported MUS were retropubic, this experience does reflect a variety of sling types (retropubic, transobturator, single-incision) and attending surgeon operative styles, adding to its heterogeneity. In our study, less experienced learners may have benefited more from the training than their more experienced counterparts. This increased impact for novice surgeons highlights the need for training opportunities outside the operative theatre. Recent studies have suggested that the number of MUS procedures performed in the US is increasing [15-16]. With the aging of the US population, the need for providers trained to perform the MUS will continue to increase [17]. MUS is the current gold-standard for surgical treatment of stress urinary incontinence, thus residency training programs should consider mastery of this procedure a necessary skill for the gynecologic generalist [18]. Educational programs such as this one provide a simple, relatively low cost, effective method for enhancing MUS training, particularly for the novice resident. Simulation can improve competence and confidence by performing the technique on a model repeatedly, which we believe will translate to less anxiety and better performance in the operating room, allowing maximum benefit from each operative experience.

Our previous research elucidated a significant amount of resident anxiety in learning to pass retropubic trocars [7]. This is understandable given the blind nature of the step and the vital structures near the path of the trocar. We hypothesized that when given the opportunity to view these anatomical landmarks on both a plastic model and a prosected cadaver in relation to the successfully placed trocar, residents would feel more confident with performing this step in the operating room. Despite encouraging results, demonstrating both subjective and objective improvement in MUS comfort and skill, our study has several limitations. Although we began with a cohort of 24 residents, only a small number attended the post-test MUS trocar model session, which significantly limits our ability to interpret our post-training data. Though we did not note any baseline differences between those who did and did not complete post-test MUS trocar model session, we cannot exclude the possibility of a differential teaching effect in these two groups. We were unable to recruit participants for the proposed interview sessions, perhaps because of the busy schedules of resident trainees, thus our qualitative data is limited. Furthermore, our follow-up time was short, limiting our ability to comment on long-term skill retention. Additionally, the interrater reliability for our checklist scores was much greater than that for the mOSATS, perhaps due to the fact that we used a scaled-back OSATS, with fewer assessment items, to facilitate the use in a filmed model situation. Further reliability and validity testing of these measures in a MUS model-based instruction is needed to determine their appropriate use.

We hope to ultimately assess resident comfort and skill with the MUS in the operative environment. Clearly, the goal of any surgical simulation training is not to simply improve task performance on a model, but to translate the acquired skill to an actual patient. Our focus on trocar passage, with less emphasis on post-operative care, may explain the lack of improvement in resident comfort with complication recognition and management. Future teaching programs may include content directed towards recognizing and managing intraoperative complications and caring post-operatively for both the routine and complicated patient.

Effective education research requires trainee participation. Despite near universal participation initially, just over half of residents returned for follow-up assessment and no residents completed the proposed short individual interviews. This discrepancy suggests both a fundamental willingness of trainees to participate in educational research and highlights the time constraints and barriers to participation inherent in a busy clinical training program. Future surgical education research projects may benefit from modified designs to reflect these limitations, such as integration of study interventions and measures into the established curriculum.

## Conclusions

We demonstrate improvement in self-reported resident comfort with the MUS procedure and improvement in skill with retropubic MUS trocar passage on a model following a novel model and cadaver-based educational program. We anticipate this improvement in comfort and skill will translate to improved intra-operative performance. Further research is needed to test the program in a live operative environment.

## Appendices

**Table 3:** Midurethral sling learner-endorsed themes

Themes: Pre-teaching	Representative resident responses
Lack of experience	*“I have no idea how to place a sling.”* *“[I] haven’t done enough to know.”*
Discomfort with potential complications	*“I wouldn’t know what to do with all the complications.”* *“[I am uncomfortable with] vaginal dissection avoiding urethra, entering correct plane with least bleeding, having enough vaginal epithelium covering mesh.”*
Discomfort with trocar passage	*“[I fear the] passage of trocars because blind procedure passing near many vital structures”* *“[I am] not comfortable that I will keep my trocar straight and not deviate.”*
Themes: Post-teaching	Representative resident responses
Praise for “hands on” nature of training	*“The teaching models were great and very informative for practicing the hands-on technique.”* *“[I enjoyed the] interactive teaching with relatively life-like models, immediate feedback while we performed procedures on the models, a variety of stations: cadaver, pelvic models, cystoscopy.”*
Importance of repetition	*“At this point I feel I just need more practice”* *“Optimal hand movements/placement [can be learned, it just] takes practice to develop muscle memory.”*
Importance of comfortable learning environment	*“Helpful to learn by experience and doing in a non-stressful situation.”* *“[I appreciated the] low-pressure learning environment.”*
